# African Trypanosomes Undermine Humoral Responses and Vaccine Development: Link with Inflammatory Responses?

**DOI:** 10.3389/fimmu.2017.00582

**Published:** 2017-05-24

**Authors:** Benoit Stijlemans, Magdalena Radwanska, Carl De Trez, Stefan Magez

**Affiliations:** ^1^Laboratory of Cellular and Molecular Immunology, Vrije Universiteit Brussel (VUB), Brussels, Belgium; ^2^Myeloid Cell Immunology Lab, VIB-UGent Center for Inflammation Research, Ghent, Belgium; ^3^Laboratory for Biomedical Research, Ghent University Global Campus, Yeonsu-Gu, Incheon, South Korea; ^4^Structural Biology Research Centre (SBRC), VIB, Brussels, Belgium

**Keywords:** B-cell lymphopoiesis, African trypanosomosis, vaccination strategies, inflammation, T-cells, macrophage migration inhibitory factor (MIF)

## Abstract

African trypanosomosis is a debilitating disease of great medical and socioeconomical importance. It is caused by strictly extracellular protozoan parasites capable of infecting all vertebrate classes including human, livestock, and game animals. To survive within their mammalian host, trypanosomes have evolved efficient immune escape mechanisms and manipulate the entire host immune response, including the humoral response. This report provides an overview of how trypanosomes initially trigger and subsequently undermine the development of an effective host antibody response. Indeed, results available to date obtained in both natural and experimental infection models show that trypanosomes impair homeostatic B-cell lymphopoiesis, B-cell maturation and survival and B-cell memory development. Data on B-cell dysfunctioning in correlation with parasite virulence and trypanosome-mediated inflammation will be discussed, as well as the impact of trypanosomosis on heterologous vaccine efficacy and diagnosis. Therefore, new strategies aiming at enhancing vaccination efficacy could benefit from a combination of (i) early parasite diagnosis, (ii) anti-trypanosome (drugs) treatment, and (iii) anti-inflammatory treatment that collectively might allow B-cell recovery and improve vaccination.

## Introduction

African trypanosomes are strictly extracellular single-celled protozoan parasites belonging to the genus *Trypanosoma*, which cause debilitating diseases in humans and livestock and consequently significantly affect the socioeconomic development of sub-Saharan Africa (1). About 70 million people distributed over a surface of one and a half million square kilometers are estimated to be at risk for contracting sleeping sickness in Africa (2). The distribution of African trypanosomes coincides mostly with the distribution of the habitat of the hematophagic insect vector, i.e., the tsetse “fly” (*Glossina* sp.), with tsetse meaning “fly” in the Tswana language of Southern Africa (3). Human African trypanosomosis (HAT) or sleeping sickness is caused by *Trypanosoma brucei gambiense* (west and central Africa) and *Trypanosoma brucei rhodesiense* (eastern and southern Africa) (4, 5). Both parasites cause infections that exhibit clinically diverse patterns and hence require different patient management, with the less prevalent *T. b. rhodesiense* HAT considered to be the more acute and virulent/lethal form of the disease (6, 7). HAT mainly affects remote rural communities where the health infrastructure is often minimal. In general, the disease is characterized by two stages: the early hemolymphatic stage whereby parasites proliferate in the blood and lymphatic system and the late meningoencephalitic stage whereby parasites penetrate the blood–brain barrier and proliferate in the cerebral spinal fluid (8). When patients in the meningoencephalitic stage remain untreated, an encephalitic reaction can occur resulting in coma and subsequent death (9–11). However, it is important to mention that in recent years a number of reports have indicated that HAT is not always lethal and that both *T. b. gambiense* and *T. b. rhodesiense* can result in chronic human infections with little or no symptoms (12, 13). Limited surveillance in particular of non-symptomatic cases, however, make it hard to assess how widespread these non-lethal cases are, or what the molecular and genetic underlying factors are that account for HAT resistance in certain individuals (14).

According to WHO, recent successes in the fight against HAT have brought the annual new cases to less than 10,000 (5, 7, 8). To design and maintain future control strategies, it is important to indicate that *T. b. gambiense* is an anthroponotic disease with a minor role for animal reservoirs that accounts for 98% of the reported HAT cases and causes a chronic, gradually progressing disease, whereby the late meningoencephalitic stage is not reached before months or even years of infection (10, 15). *T. b. rhodesiense* on the other hand is a zoonotic disease affecting mainly animals (livestock and wildlife), with humans being only accidentally infected, and represents only 2% of the reported HAT cases, whereby the infections are acute and progress rapidly (within weeks) to the late meningoencephalitic stage (10, 16). The zoonotic nature of *T. b. rhodesiense* infections make them more difficult to control compared to *T. b. gambiense* infections (15, 17, 18). Animal African trypanosomosis (AAT) also known as Nagana is a second form of trypanosomosis that affects sub-Saharan Africa. It is mainly caused by *Trypanosoma congolense, Trypanosoma vivax*, and to a lesser extent *Trypanosoma brucei brucei*, while surra and dourine are also forms of AAT caused by *Trypanosoma evansi* and *Trypanosoma equiperdum*, respectively (19–21). Of note, some parasites acquired a mechanical transmission mode (hence, they can reside outside the tsetse/vector belt) and are also found in South/Latin America (*T. vivax* and *T. evansi*) and Asia (*T. evansi* and *T. equiperdum*) (19, 21–23). Yet, *T. congolense* forms a major constraint on livestock production and remains the leading cause of livestock morbidity and mortality in sub-Saharan Africa. Hereby, cattle succumb to infection primarily due to parasite-induced anemia or complications resulting from secondary, opportunistic infections (24). Progressive disease for a prolonged time will weaken these animals, thereby preventing them to be used as draft animals or for food/milk production. As a result, farming in the tsetse belt remains challenging and hampers the development of poor societies, leading to great economic losses in terms of productivity (25, 26). Indeed, AAT accounts for an estimated annual loss of about US$5 billion, whereby Africa invests every year at least US$30 million to control cattle trypanosomosis in term of curative and prophylactic treatments (27, 28). The total losses for the total tsetse-infested lands in terms of agricultural gross domestic product are US$4.75 billion per year (1). In fact, the impact of AAT on the affected areas is the combined result of environmental, political, sociocultural, entomological, and livestock management factors (29), whereby (i) the political instability of the areas hampers controlled intervention strategies and subsequently discourages commercial investment in control strategies, (ii) pharmaceutical companies are less prone to engage/invest in drug discovery/development against diseases that affect the poorest people, (iii) wild animals function as reservoir of the parasite and therefore hamper the control of the disease, and (iv) the inappropriate use of the available drugs resulting in the emergence of drug resistance (30, 31). Up till now, not a single-field applicable vaccine exists, and chemotherapy is the only strategy available to treat the disease, which is associated with high drug toxicity. Nevertheless, so far chemotherapy remains the only therapeutic choice for these diseases, whereby they target unique organelles of trypanosomes such as glycosomes and the kinetoplast that are absent in the mammalian host or trypanosome metabolic pathways that differ from the host counterparts [carbohydrate metabolism, protein and lipid modifications, and programmed cell death (PCD)] (32–34). Unlike the situation with HAT, where the nifurtimox–eflornithine combination therapy is the preferred first-line treatment for second-stage disease (35, 36), no drug combinations are currently used for AAT (27). Instead, alternating use of compounds, particularly diminazene and isometamidium (called a “sanative pair”), with low risk of cross-resistance, is recommended where possible. Hence, there is an urgent need to optimize trypanocide usage/delivery such as extending the half-life of current trypanocides to use lower quantities of trypanocide in a more effective way and, consequently, pose a decreased risk of toxicity and possibly decreased resistance development (37, 38). However, there is some optimism since progress in HAT/AAT control measures were made over the past decade due to the establishment of the Pan-African tsetse and trypanosome eradication campaign, funded by the African Development Bank, which was established in the year 2000. This organization has set tsetse elimination as its goal and has strengthened renewed interest in the research and development of control/intervention options (29, 39). Overall, “elimination” of *T. b. gambiense* HAT has been targeted for 2020 under leadership of the WHO (40).

One crucial factor that stands in the way of total eradication of trypanosomosis in general is inefficient diagnosis of the infection. To date, microscopy detection of the parasite remains the only available tool to diagnose AAT and *T. b. rhodesiense* HAT in a reliable way. Only for *T. b. gambiense*, monitoring tools are available for both detection of exposure and staging of the disease (41). The latter is important to reduce the risk of treatment-associated complications occurring during treatment of the second stage of the disease (42). In this context, improvement in staging diagnosis and early screening methods are current challenges, which would avoid delayed patient treatment. Diagnosis is often hampered due to lack of positive predictive value of existing field applicable techniques and the fact that antibody (Ab)-based detection cannot differentiate between active or passed—but cured—infections (41). Immunodiagnostics based on antigen detection in this case would be preferable but are currently non-existent for trypanosomosis in the field (41, 43). An additional complication resides in the recent finding that tsetse-transmissible *T. b. gambiense* parasites can be found in human skin biopsies from undiagnosed individuals (44). Hence, this suggests that the current diagnostic methods and control policies need to be reevaluated.

In the next sections, we will give an overview of (i) the different escape mechanisms used by African trypanosomes to survive within their mammalian host and (ii) their strategies to undermine the entire host immune response, including the humoral response, which in turn hampers vaccine development. This review will focus on two most relevant AAT species *T. brucei* and *T. congolense*, given that for both parasites, established murine models and field studies in the economically and clinically relevant host (cattle) are available (45). While for *T. vivax*, field study information is scanty, hardly any representative experimental data are available as these parasites do not grow in mice unless they are carefully adapted (23, 46, 47). However, there is prospect since Minoprio and coworkers were able to establish a murine model for *T. vivax* (48). Although rodents are not natural hosts for these pathogens, murine models can be considered valuable tools to unravel the interactions and the immune evasion mechanisms of these parasites with their mammalian host.

## Host–Parasite Interactions

### Life Cycle of African Trypanosomes

African trypanosomes have a digenetic/heteroxenous complex life cycle alternating between the intestine of the tsetse fly vector and the blood/tissues of the mammalian host, whereby they progress through different developmental stages, i.e., procyclic or trypomastigote forms, respectively (49, 50). Yet, to survive in each of these hosts, they undergo essential changes at the level of morphology, energy metabolism, and surface coat protein expression (51, 52). Hereby, trypanosomes feed by absorbing nutrients (proteins, carbohydrates, and fats) as well as iron and oxygen from the body fluids of the host to generate the energy necessary for the vital processes (53). Within the bloodstream of the mammalian host, they subsist as bloodstream forms (BSFs) that are ingested by tsetse flies during a blood meal, wherein they differentiate into procyclic forms in the insect midgut. Next, they migrate to the proboscis (mouth parts) where they differentiate into epimastigote forms and finally into infective metacyclic forms (MCFs) that can be transmitted to a new mammalian host during the next blood meal. Although within the tsetse fly both *T. brucei* and *T. congolense* parasites have a similar migratory life cycle (i.e., initial establishment of midgut infection and invasion of the proventriculus), they exhibit differences in transitional developmental stages with production of infective MCFs in the proboscis for *T. congolense* and in the salivary glands for *T. brucei* (54–56). Within the mammalian host, *T. congolense* is a strictly intravascular parasite, whereby they bind to circulating erythrocytes and endothelial cells through their flagellum, causing damage at the adhesion site (57, 58). In contrast, *T. brucei* can also extravasate blood vessels and invade tissues and cause severe tissue injury (44, 59, 60). Hence, this implies differences in virulence mechanisms, host–pathogen relationships, and pathogenic effects between the two species (61). In addition, *T*. congolense exists strictly as a long slender (LS) dividing form, whereas *T. brucei* parasites are pleomorphic (i.e., can exhibit two forms); a LS dividing form and a short stumpy (SS) non-dividing form that is preadapted for transmission to the fly (62, 63). This transition, which involves a quorum sensing factor (i.e., an enigmatic stumpy inducing factor), is suggested to help control parasitemia and to increase the host survival time, thereby increasing the probability for successful transmission of the trypanosomes to a new host (50, 64). It is suggested that this removal of the majority of the population is an altruistic form of PCD and the counterpart of apoptosis in metazoan (65, 66). Hereby, increased intracellular reactive oxygen species and prostaglandin D2, which is produced principally by stumpy forms, are promoting this PCD that can be considered as a second control point in terminal differentiation to the SS form (67, 68). Moreover, it is suggested that the SS form is heterogeneous, whereby one part is altruistic and undergoes apoptosis-like events, thereby stimulating the host’s immune response and eliminating the major LS and SS antigen population, while the other is tsetse infective (69). Both the LS and SS forms are covered by a dense variant surface glycoprotein (VSG) coat, which protects them from both the innate and the adaptive host immune systems. Of note, within the tsetse fly, the parasites are covered by a procyclin coat, and only when they differentiate into the MCF (infective form), they express a metacyclic VSG coat (70).

### Parasite Escape Mechanisms in the Mammalian Host

To survive as extracellular parasites within the mammalian host environment (i.e., blood or extravascular tissues), African trypanosomes have developed efficient immune evasion mechanisms, at both the parasite level and the level of modulating host responses. Indeed, during millions of years of coevolution with their mammalian host, these parasites have “learned” to divert and sculpture the host immune system to prevent the generation of an effective response. The most predominant changes at the level of the host occurring during African trypanosomosis are massive splenomegaly coinciding with destruction of the lymphoid architecture and hepatomegaly. These modulations are followed by lymphadenopathy and hypergammaglobulinemia, leading to systemic multiple organ failure and death in experimental mouse models (71). In this section, we will give an overview of the most prominent escape mechanisms trypanosomes developed to allow successful infection within their mammalian host.

#### Parasite-Associated Escape Mechanisms

**Figure 1:**
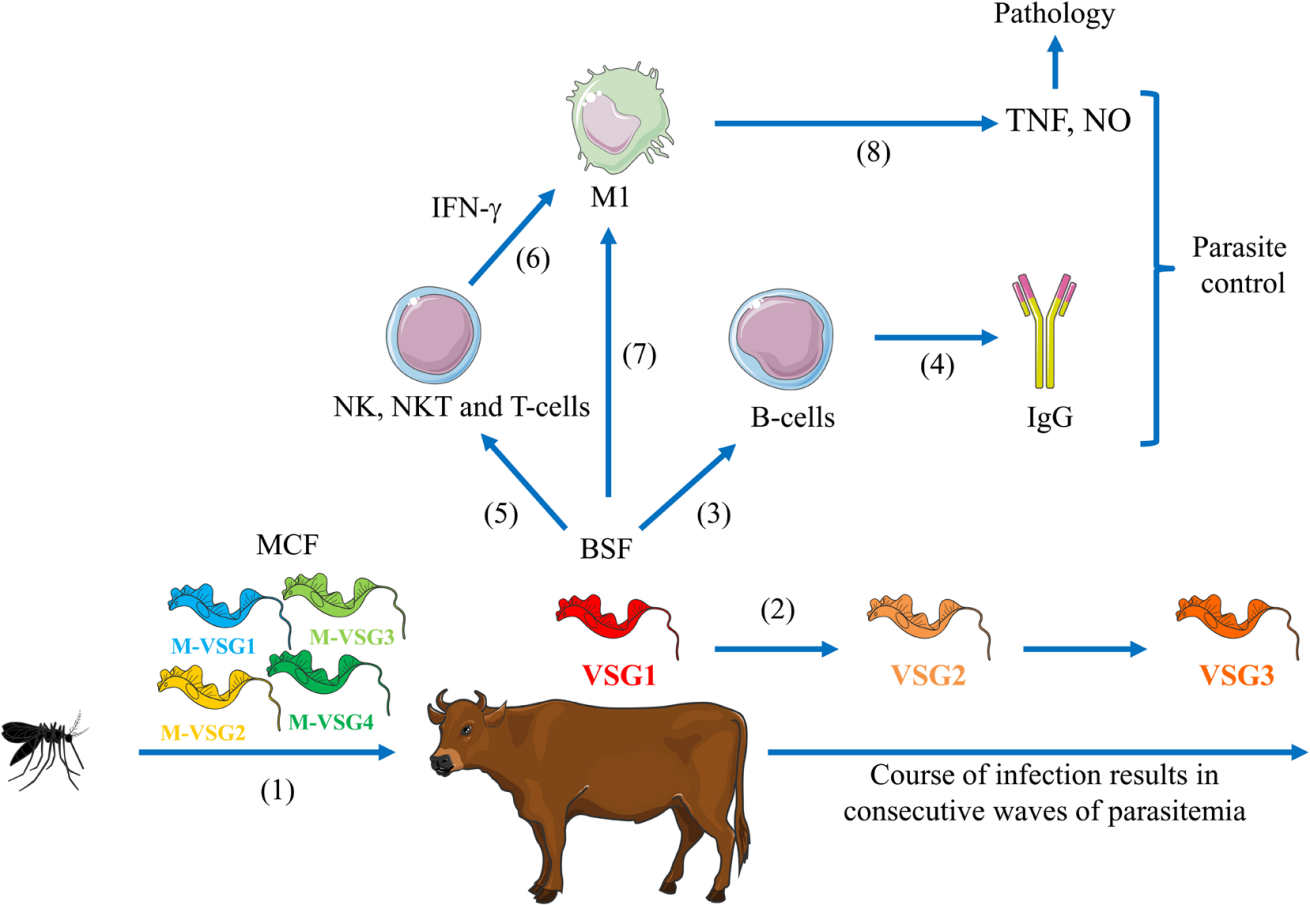
**Model for African trypanosomosis within the mammalian host**. (1) Upon the bite of a trypanosome-infected tsetse fly, metacyclic form (MCF) parasites are inoculated within the mammalian host. (2) These parasites differentiate into bloodstream forms (BSFs) and switch their metacyclic VSG (M-VSG) into a bloodstream uniform VSG (VSG1) giving rise to the first parasitemia peak. During the course of infection, there is antigenic variation (VSG2, VSG3, etc.) giving rise to different peaks of parasitemia. (3,4) Parasites-derived components trigger B-cell activation and production of antiparasite IgG needed for parasitemia control. (5,6) Parasite-derived components trigger NK, NKT, and T-cell activation resulting in the production of interferon-gamma (IFN-γ). (7) Parasite-derived components in concert with IFN-γ trigger the induction of classically activated macrophages (M1 cells). (8) These M1 cells release pro-inflammatory cytokines [like tumor necrosis factor (TNF)] that are needed for parasite control, but at the same time contribute to pathology development if maintained during the course of infection.

Already at the onset of infection, i.e., inoculation of trypanosome-containing saliva upon the bite of a tsetse fly, components present in the saliva are able to (i) dampen local host inflammatory immune responses characterized by the release of trypanolytic molecules, i.e., tumor necrosis factor (TNF) and nitric oxide (NO), thereby favoring parasite development and (ii) trigger mast cell degranulation resulting in release of histamine and increased vasodilatation, thereby allowing parasite dissemination/extravasation into the blood circulation [reviewed in Stijlemans et al. (72)]. Within the mammalian host, trypanosomes are very proficient in avoiding and subsequently reorchestrating host immune responses. Being extracellular parasites, they are confronted with the host’s humoral immune response; hence, to allow infection to occur, they have to overcome this major obstacle. In first instance, these parasites are covered with a very dense coat composed of approximately 5 × 10^6^ identical VSG homodimers of 50–60 kDa subunits that are anchored in the plasma membrane by a glycosylphosphatidylinositol (GPI) anchor, which functions as a ~15-nm thick barrier and protects the cell from Abs that might bind to buried conserved proteins (73, 74). Second, to prevent Ab-mediated elimination by Abs raised against the immunodominant/immunogenic VSGs, these parasites acquired a system of antigenic variation, whereby they are equipped with a battery of more than 1,000 different VSG genes and pseudogenes in their genome that in turn can undergo segmental gene recombination to encode an estimated 10,000 different VSG surface coats during infection (75). Hence, at regular time points [i.e., upon recognition by the host’s humoral response or when a maximal density is reached (Quorum sensing)], they switch their coat into a different variable antigen type, thereby allowing escaping Ab-mediated elimination (76). This antigenic variation is accomplished by (i) *in situ* switching of transcriptional control (i.e., changing the VSG expression site) or (ii) gene replacement resulting in a switch of the terminal telomeric VSG gene itself (77, 78). Besides antigenic variation, these parasites were shown to express a mosaic VSG during the process of VSG switching (i.e., changing from metacyclic to BSFs, or during the course of infection), which in turn might be an efficient way to prevent effective Ab recognition (79, 80). Also the infective MCFs use this differential VSG expression to generate diversity and counter existing partial immunity/enhance transmission, while BSF use this to prolong infection (see Figure 1). Interestingly, the MCFs initiate VSG expression by each cell, activating at random one from a small subset of metacyclic VSG (M-VSG) genes, resulting in a heterogenous population, whereby each trypanosome expresses a single VSG (81, 82). Hereby, the M-VSG expression is regulated exclusively at the transcriptional level, while the bloodstream VSG expression is regulated mainly at the posttranscriptional levels and transcribed polycistronically (83). Third, trypanosomes also exhibit a very high endocytosis rate as an efficient way to acquire nutrients and at the same time to remove Ab-bound VSG molecules and thereby prevent Ab-mediated or even complement-mediated opsonization/elimination (84, 85). This might allow parasites to transiently escape T-cell-independent B-cell-mediated elimination, which is the first line of defense. This immunological escape also gives time to transform into trypomastigote forms, which are adapted to survive in the mammalian host during the initiation of infection and gives the parasites an immunological advantage during the process of antigenic variation.

Although mainly IgMs play a key role during *T. brucei* infection, while in *T. congolense* infections, IgGs are mostly important (86, 87), it could be assumed that complement-mediated lysis is also an important innate defense mechanism. However, trypanosomes have developed efficient mechanisms to avoid complement-mediated elimination. First, trypanosomes are able to avoid elimination *via* the complement pathway, which is typically activated *via* immune complexes with Abs. Indeed, by releasing vast amounts of soluble VSG (mainly at the peak of parasitemia), Abs and complement factors will be scavenged and thereby induce a state of hypocomplementemia that can favor the survival of the parasites (88). Second, besides undermining the classical activation of the complement pathway that could contribute to trypanosome clearance through Ab-mediated trypanolysis and/or phagocytosis, the alternative pathway of complement activation occurring in the absence of specific Abs (i.e., during early stages of infection) is also impaired. Indeed, by masking sites on the VSG plasma membrane, which are capable of promoting alternative pathway activation, the cascade is blocked at the C3 convertase stage, thereby impairing the generation of the terminal complex (C5–C9) that normally induces trypanolysis (89, 90). However, it seems that the later stages of the complement activation cascade do not play a detrimental role in parasite control. Indeed, in AKR mice, which are natural C5 KO mice (91), the absence of the complement lysis pathway does not prevent periodic trypanosome clearance and does not hamper long-term survival in case of *T. congolense* infections (92, 93). However, soluble complement molecules, such as C3a and C5a, secreted during early stages of trypanosome infection, can contribute to the initiation of the early inflammatory immune response and also act as (i) chemotactic agents attracting phagocytes to the site of infection and (ii) release histamine from mast cells, thereby increasing microvascular permeability (94), which would allow/enable parasite extravasation into the blood circulation.

#### Parasite-Induced Escape Mechanisms

Besides being equipped with tools to avoid elimination by the host’s “innate” humoral response, trypanosomes also undermine the host cellular immune responses to allow chronic infection. Moreover, the data in literature suggest that the efficiency to modulate the innate immune response is crucial for the progression of trypanosomosis (95). Hereby, the suppression of cellular immune responses is an efficient mechanism to evade host defense mechanisms and a general feature of trypanosomosis in bovine, human, and murine hosts. To this end, these parasites are equipped with a battery of molecules able to modulate early antiparasite responses to allow establishment. It is important to mention that the course of an African trypanosome infection can be characterized by an early release of interferon-gamma (IFN-γ) by activated NK-, NKT- and T-cells required to induce classically activated macrophages (M1) (see Figure 1). In turn, these activated M1 develop upon exposure to parasite-derived molecules such as VSG and CpG a type-1 inflammatory immune response leading to the production of the potential trypanocidal molecules such as TNF and NO that in conjunction with Abs will contribute to parasite control (96, 97). Yet, persistence of this type-1 immune response and hyperactivated M1 cells will culminate in trypanosusceptible animals into immunopathological features such as the systemic immune response syndrome and anemia (98). Trypanotolerant animals on the other hand are able to switch to a more type-2 immune response and the induction of alternatively activate macrophages (M2), whereby the anti-inflammatory cytokine interleukin (IL)-10 was shown to play a pivotal “dampening” role (99, 100).

##### Undermining Macrophage Functionality

To sustain the development of the first (most prominent) peak of parasitemia in the blood and its control by the host, some parasite-derived molecules are able to dampen pro-inflammatory responses (TNF, NO) by these M1. Most research so far has been performed using *T. brucei* infections and indicate that these parasites release components such as adenylate cyclase (AdC) and kinesin heavy chain (TbKHC-1) to dampen initial host responses, thereby allowing early parasite establishment (101, 102). Indeed, AdC released by altruistic parasites upon parasite phagocytosis by liver-associated myeloid cells prevents production of the trypanolytic cytokine TNF (*via* a protein kinase A pathway), which promotes early establishment of trypanosomes within the mammalian host (101). On the other hand, the release of TbKHC-1 by parasites induces IL-10 and arginase release by myeloid cells in a SIGN-R1-dependent manner and favors initial parasite seeding by inducing the production of polyamines, which constitute trypanosome essential nutrients (102, 103). Recently, it was also shown that metabolites produced by trypanosomes such as indolepyruvate (i.e., a transamination product of tryptophan) can dampen macrophage pro-inflammatory responses that prevent elimination (104). Finally, the order of exposure to parasite-derived versus host-derived macrophage-activating components as well as the relative concentration of these mediators may influence the ability of the host to respond to trypanosome infections. Indeed, early during infection, exposure of macrophages to soluble VSG (encompassing the glycosylinositolphosphate substituent) before IFN-γ priming downregulated the level of signal transducer and activator of transcription 1 phosphorylation, which in turn reduced transcription of pro-inflammatory cytokines such as TNF (105). So far, nothing is known about such mechanisms for *T. congolense*. In summary, it seems that trypanosomes have developed a system whereby altruistic parasites are phagocytosed, thereby disabling the M1-mediated innate immune response required for parasite control and paving the way for initiation and establishment of the first wave of parasitemia.

##### Modulation of T-Cell Functionality

Besides undermining the antitrypanosomal potential of the myeloid system, the parasite is also impairing T-cell help required to mount a more efficient response during the course of infection. Early during *T. brucei* and *T. congolense* infection, T-cell suppression is occurring *via* suppressive myeloid cells by inhibiting IL-2 secretion and downregulation of IL-2 receptor expression (106–108), whereby prostaglandins were found to play an important role in the murine model, but not in the bovine model (109). In addition, early data on T cell regulation and trypanosomosis showed that both IFN-γ and TNF play a key role in the suppressive effects on CD4 and CD8 T-cells (107). Furthermore, this suppressive phenotype of the host cells during the early stages of *T. brucei* infection is due to a combination of (i) trypanosome-released macrophage-activating factors leading to secretion of immunosuppressive factors such as NO, prostaglandins, and TNF and (ii) host-derived IFN-γ needed for optimal macrophage activation (110, 111). Moreover, this work also showed that within the *T. brucei* model, there is a compartmentalization of the suppressive effect in murine models during the later stages of infection, whereby NO plays a key role in macrophage-mediated splenic suppression, whereas the macrophage-mediated lymph node suppression occurred in an IFN-γ-dependent manner (110). Hence, at this stage of infection, an IFN-γ-independent suppressive mechanism is elicited in the spleen, whereas in the lymph nodes, IFN-γ is required yet not sufficient to inhibit T cell proliferation. In this context, it was shown that the trypanosome suppression-inducing factor (TSIF) released by *T. brucei* during the course of infection induces TNF and NO secretion by classically activated macrophages (i.e., M1), which is a prerequisite for parasite control. However, at the same time, it blocks T-cell proliferation in a NO, IFN-γ, and cell contact-dependent manner as well as downregulates type-2 immune responses required to dampen M1-mediated pathogenicity (112). Moreover, TSIF was shown to be essential for parasite biology given that TSIF knock-down parasites die within 2 days. In the *T. congolense* model, it was shown that besides IFN-γ, the anti-inflammatory cytokine IL-10 also contributed to T-cell suppression (113). Finally, although both murine and bovine African trypanosomosis induce suppression, it seems that NO does not play a role in the loss of T-cell proliferative function in the bovine trypanosomosis model and that, in contrast to the mouse model, the capacity of monocytes and macrophages to produce NO is actually downregulated in infected cattle (114). In summary, these results suggest that the T-cell suppression is multifactorial, tissue and infection stage dependent, and host/parasite dependent. However, T-cells are dispensable for parasite control, which was evidenced by the fact that mice lacking a functional T-cell compartment are as efficient as immune-competent animals in controlling trypanosome infection (86). These data indicate that T-cell-independent B-cell-mediated elimination is the driving factor implicated in controlling parasitemia. Nevertheless, T-cells play a key role in the development of African trypanosomosis-associated pathogenicity, such as anemia (115).

##### Undermining B-Cell Functionality

Given that trypanosomes are extracellular parasites, it is not surprising that the host–parasite coevolution resulted in a subtle equilibrium between suppression of B-cell and Ab functionality and parasite persistence. Indeed, besides suppression of myeloid cells and T-cells, B-cells were also found to be negatively affected during the early stages of trypanosome infection. Accordingly, trypanosomes exert full control of the different types of host immune responses to establish chronic infection. In this context, in cattle, it was found that there are also differences in humoral responses between *T. congolense*-infected trypano-resistant (N’Dama) and trypanosusceptible Boran cattle, further highlighting the importance of the humoral immune response in parasitemia control (116). In the following section, the effect of African trypanosome infections at the level of the B-cell compartment will be scrutinized.

**Figure 2:**
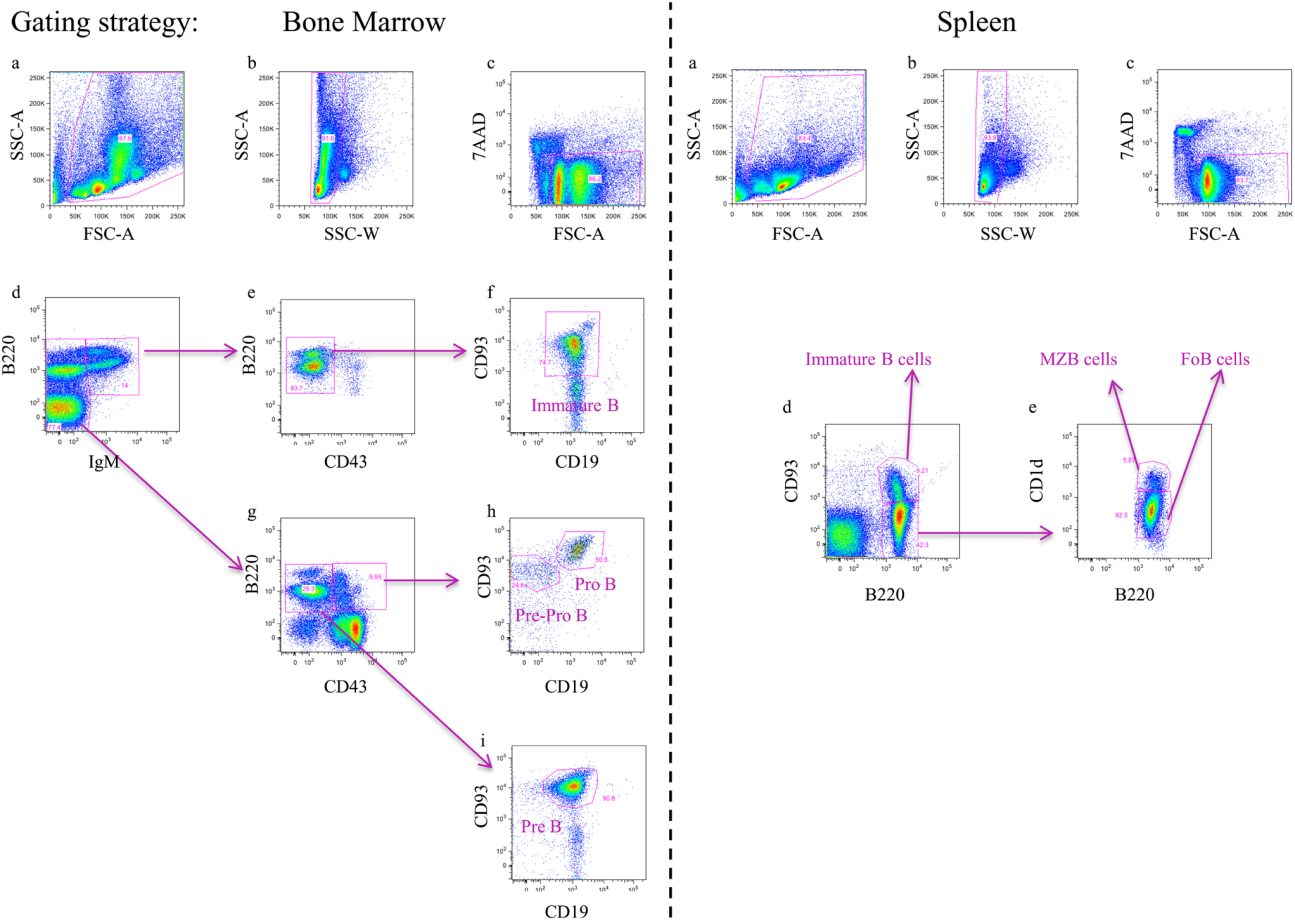
**FACS gating strategy to track B-cell alterations during infection within the bone marrow (BM) and spleen**. BM (left panel): **(A,B)** In an FSC-A versus SSC-A plot, a life gate was selected followed by gating on singlets in an SSC-A versus SSC-W plot, respectively. **(C,D)** Following gating on 7AAD^−^ cells and plotting in a B220 versus IgM allow identification of IgM^+^ and IgM^−^ cells. **(E,F)** The IgM^+^ cells are plotted in a B220 versus CD43 plot to identify B220^+^CD43^−^ cells and subsequently plotted in a CD93 versus CD19 to identify immature B-cells (CD93^+^CD19^+^). **(G)** The IgM^−^ cells are plotted in a B220 versus CD43 plot to identify B220^+^CD43^−^ cells and B220^+^CD43^+^ cells. **(H)** The B220^+^CD43^−^ cells are plotted in a CD93 versus CD19 to identify pre B-cells (CD93^+^CD19^+^). **(I)** The B220^+^CD43^+^ cells are plotted in a CD93 versus CD19 to identify pro B-cells (CD93^+^CD19^+^) and pre-pro B-cells (CD93^+^CD19^−^). Spleen (right panel): **(A,B)** In an FSC-A versus SSC-A plot, a life gate was selected followed by gating on singlets in an SSC-A versus SSC-W plot, respectively. **(C,D)** Following gating on 7AAD^−^ cells and plotting in a CD93 versus B220 allow identification of immature B-cells (CD93^+^B220^+^). **(E)** The CD93^+^B220^−^ cells are subsequently plotted in a CD1d versus B220 plot to identify marginal zone B (MZB) cells (CD1d^+^B220^+^) and follicular B (FoB) cells (CD1d^−^B220^+^). It is important to mention that during infection, the expression levels of typical splenic B-cell subset markers can be modulated, which substantially complicates the identification of the different B-lymphocyte subsets.

**Figure 3:**
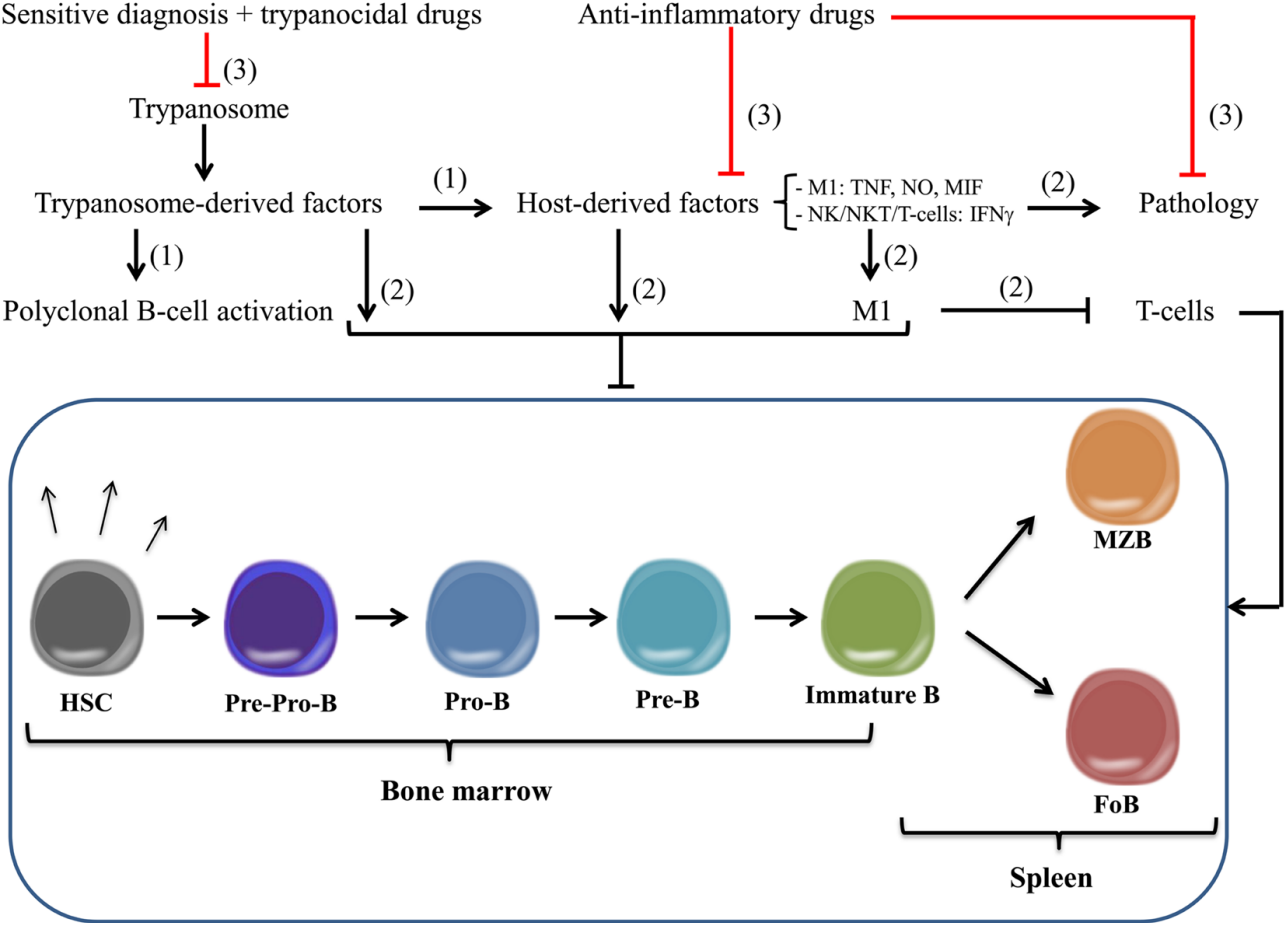
**Model for African trypanosomosis-associated impaired B-cell lymphopoiesis and improved vaccine development**. (1) During the course of African trypanosomosis (AT), parasite-derived components are released that trigger besides polyclonal B-cell activation also the production of host-derived pro-inflammatory factors (i.e., NK-, NKT-, and T-cell-derived IFN-γ, M1-cell-derived TNF, NO, MIF) needed directly/indirectly for early parasite control. Yet, following control of the first parasitemia peak, polyclonal B-cell activation leads to dilution of parasite-specific antibodies, whereas the persistent pro-inflammatory response contributes to suppression of host responses and pathology. (2) Both parasite- and host-derived components can lead to a general state of impaired B-cell lymphopoiesis in (i) the bone marrow (BM), ranging from pre-pro-B-cell, pro-B-cell, pre-B-cell, and immature B-cell and (ii) the spleen, ranging from immature B-cell till MZB cell and FoB cell. In addition, also host-derived factors (involving M1) can contribute to T-cell suppression that in turn can affect B-cell homeostasis. (3) Hence, a more efficient therapeutic intervention strategy for AT should consist of a combination of (i) more reliable/sensitive diagnosis systems allowing early-stage parasite detection, (ii) more efficient trypanocidal/toxic drugs allowing improved parasite treatment, and (iii) pro-inflammatory-blocking molecules that could lead to a reduced pathology and a restoration of normal B-cell responses, thereby allowing more efficient/optimal vaccination. M1, classically activated myeloid cells; MIF, macrophage migration inhibitory factor; IFN, interferon; TNF, tumor necrosis factor; NO, nitric oxide; MZB, marginal zone B; FoB, follicular B.

In homeostatic conditions, B-cells develop from bone marrow (BM)-derived hematopoietic stem cells (HSCs) that initially differentiate into multipotent progenitor cells and subsequently into common lymphoid progenitor cells (117). Next, B-cell lymphopoiesis occurs through several developmental stages, such as pre-pro-B, pro-B, pre-B, and, finally immature B-cells, which is a highly regulated process with alternating phases of cell proliferation and differentiation (118, 119). During this process, these different B-cell subsets rearrange their immunoglobulin heavy-chain and light-chain gene loci and express different surface markers that can be identified *via* flow cytometry (see Table 1; Figure 2, left panel). Within the BM, these B-cells also undergo a positive and negative selection procedure, whereby the B-cell receptor (BCR) plays a checkpoint role (120). If the BCRs do not bind their antigen, they stop their development, i.e., during positive selection, while during negative selection, binding of self-antigens to the BCR triggers either clonal deletion, receptor editing, anergy, or ignorance, resulting in central tolerance (121). At the last stage of differentiation within the BM, these immature B-cells exhibit a high IgM expression and low or no expression of the IgD maturation marker. To complete their development, immature B cells migrate to the spleen *via* the blood as transitional B cells (T1 type). In the spleen, these transitional B (T1) cells differentiate into T2 cells before they develop into two types of mature naïve B-cells (122), namely follicular B (FoB) or marginal zone B (MZB) cells (see Figure 2, right panel). In homeostatic conditions, B (FoB) cells are mainly located in the white pulp area of the spleen where they form primary B-cell follicles, preferentially undergo T-cell-dependent activation (upon activation *via* proteins and glycoproteins), and can give rise to both short-lived plasma cells (i.e. plasmablasts) for immediate protection and high-affinity class-switched IgG long-lived plasma cells and memory B cells for persistent protection. In contrast, MZB cells are concentrated outside the splenic marginal sinus surrounding the white pulp. Most of the time, they initiate a fast and preferentially T-cell-independent activation (upon activation *via* polysaccharides or unmethylated CpG DNA) giving rise to not only short-lived plasma cells that rapidly produce low-affinity Abs of IgM isotype but also some populations of long-lived plasma cells (123). Overall, B-cell activation is considered a very efficient defense system against invading “extracellular/blood-borne” pathogens. However, African trypanosomes have developed efficient ways to undermine the host’s humoral response to establish chronic infection and allow completion of its life cycle/transmission. Indeed, using murine models, it was shown that African trypanosomes (both *T. brucei* and *T. congolense*) already during the early stages of infection trigger polyclonal B-cell activation in an attempt to dilute-out VSG-specific Abs during the course of infection. For example, it was shown that the CpG motifs of the *T. brucei* trypanosomal genomic DNA triggers TLR-9 signaling events and contributes to polyclonal B-cell activation (96). This phenomenon might contribute to parasite immune evasion by driving unselective differentiation of B cells into short-lived plasma cells. In addition, Fcγ-receptors on phagocytes become saturated by polyspecific Abs, thereby reducing the efficiency of opsonization-mediated parasite clearance. Besides polyclonal B-cell activation, trypanosomes also undermine the “protective” humoral response by ablating B cell lymphopoiesis in primary and secondary lymphoid organs during both *T. brucei* and *T. congolense* infections. This was reflected by a depletion of all developmental B-cell stages in the BM and the spleen as well as previous effector B cells, such as memory B cells (124–126), thereby preventing the development of a B-cell memory required for permanent elimination (Figure 3). Similar results were obtained in the experimental *T. vivax* model (127). In addition, experimental results obtained in mice and livestock animals have shown that trypanosome infections exert detrimental effects on non-pathogen-related vaccines, by preventing the occurrence of memory recall responses (126, 128–130), or on the maintenance of the antigen-specific plasma B cell pool driving the development of collagen-induced arthritis (CIA) in DBA/1 prone mice (131). This destruction of the B-cell compartment at the level of both the BM and the spleen could be attributed to either parasite-derived components and/or host-derived (infection-induced) components. Interestingly, a recent work by Cnops et al. (132), revealed that during murine infection with a chronic low-virulent *T. b. gambiense* field isolate, FoB cells are retained, which coincided with reduced production of TNF and IFN-γ pro-inflammatory cytokines during the acute stage of infection compared to *T. brucei* and *T. congolense* infections. This finding was paralleled by the finding of Lejon et al. (133), which showed that in *T. b. gambiense*, HAT patients’ low parasite levels seem to be associated with limited B cell dysfunction, whereby B-cell memory responses are only slightly reduced; however, the functionality of these memory B cells was not verified in rechallenge studies. These findings indicate that in both experimental trypanosomosis and natural infection, the inflammation stage linked to the acuteness of infection could be a major determinant in the processes that drive B-cell compartment destruction. This hypothesis is reinforced by the fact that both IFN-γ^(−/−)^ and IFN-γR^(−/−)^ mice are protected from early trypanosomosis-associated FoB cell depletion (134). This phenotype coincided with a drastic inhibition of B-cell apoptosis and a reduced activation of FoB cells and inflammatory responses during the first week postinfection. These data demonstrated that IFN-γ is an important cytokine involved in undermining trypanosomosis-associated B-cell responses. So far, the cellular source of early IFN-γ production involved in triggering impaired B-cell lymphopoiesis remains to be fully elucidated. However, it was recently suggested that NK-cells, an important early source of IFN-γ, are involved in B-cell killing and suppressing humoral immunity within the *T. brucei* model (115, 135). Yet, it cannot be excluded that other sources of IFN-γ, such as NKT, CD8^+^, and CD4^+^ T-cells, are also involved in IFN-γ-mediated B-cell apoptosis given that there is a transition of IFN-γ production by these cells during the course of *T. brucei* infection (115). Interestingly, upon drug treatment [suramin and diminazene aceturate (Berenil)] of *T. brucei*- and *T. congolense*-infected mice, the BM B-cell lymphopoiesis is reinitiated, and the splenic B-cell subsets are repopulated, suggesting that an active chronic infection (i.e., parasite–host interaction) is involved in undermining the humoral responses *via* either parasite-released components and/or inflammatory-based mechanism(s) (136, 137). However, Uzonna and coworkers (137) showed that Berenil besides exerting trypanolytic effects could also modulate the host immune response to the parasite by dampening excessive immune activation and production of pathology-promoting pro-inflammatory cytokines. Hence, it cannot be excluded that the beneficial effects of Berenil for treatment of AT are multifactorial: (i) eliminate parasites thereby resulting in reduced triggering of host inflammatory immune responses and (i) reduce the host’s pro-inflammatory potential to respond to pro-inflammatory/parasite-derived components. In both cases, this will result in a more efficient host-mediated parasite control due to a recovery from the impaired B-cell lymphopoiesis and protection from infection-associated pathogenicity due to lower inflammatory responses (138). In this context, it was recently shown within the *T. congolense* experimental model that also host molecules such as macrophage migration inhibitory factor (MIF) can play a key role in regulating trypanosomosis-associated pro-inflammatory responses and B-cell homeostasis (139). In this work, it was shown that *T. congolense*-infected MIF-deficient mice exhibited increased Ab titers that correlated with reduced B-cell apoptosis. Hence, MIF could be considered as a target to alleviate the impaired B-cell lymphopoiesis.

**Table 1:** **B-cell surface marker expression used to track cellular alterations during infection**.

	Marker expression
Hematopoietic stem cell (Lin^−^)	(Terll9, CD3, CDllb, GR1, NK1.1)^−^, B220^+^, CD93^+^, IL7r, ckit^+^, CD34^+^
Common lymphoid progenitor (Lin^−^)	(Ter119, CD3, CD11b, GR1, NK1.1)^−^, B220^+^, CD93^+^, IL7r^+^, ckit^+^, CD34^−^
Pre-proB (Lin^−^)	(Ter119, CD3, CD11b, GR1, NK1.1)^−^, B220^+^, CD93^+^, CD19^−^, IgM^−^, CD43^high^
ProB (Lin^−^)	(Ter119, CD3, CD11b, GR1, NK1.1)^−^, B220^+^, CD93^+^, CD19^+^, IgM^−^, CD43^high^
PreB (Lin^−^)	(Ter119, CD3, CD11b, GR1, NK1.1)^−^, B220^+^, CD93^+^, CD19^+^, IgM^−^, CD43^low/−^
Immature B (BM) (Lin^−^)	(Ter119, CD3, CD11b, GR1, NK1.1)^−^, B220^+^, CD93^+^, CD19^+^, IgM^+^, CD43^low/−^
Transitional B (Lin^−^) (blood/spleen)	(Ter119, CD3, CD11b, GR1, NK1.1)^−^, B220^+^, CD93^+^, CD19^+^, IgM^+^, IgD^+^, CD21^+^
Immature B (spleen) (Lin^−^)	(Ter119, CD3, CD11b, GR1, NK1.1)^−^, B220^+^, CD93^+^, CD19^+^, IgM^+^, IgD^+^, CD21^+^
Mature B	(Ter119, CD3, CD11b, GR1, NK1.1)^−^, B220^+^, CD93^−^, CD19^+^, IgM^+^, IgD^+^, CD21^+^
Marginal zone B	(Ter119, CD3, CD11b, GR1, NK1.1)^−^, B220^+^, CD93^−^, CD19^+^, IgM^+^, IgD^+^, CD21^+^, CD1d^high^
Follicular B	(Ter119, CD3, CD11b, GR1, NK1.1)^−^, B220^+^, CD93^−^, CD19^+^, IgM^+^, IgD^+^, CD21^+^, CD1d^−^

## Conclusions and Perspectives

The extracellular African trypanosomes have acquired efficient immune evasion mechanisms to undermine protective host immune responses and allow survival in the host’s extracellular environment. Hereby, they are proficient in avoiding elimination *via* the host’s humoral immune response by destroying the B-cell compartment/memory and shielding-off conserved epitope, thereby paving the way for chronic infection. This destruction of B-cell memory already very early during infection might explain the failure of developing an effective vaccine. Indeed, immunization with the immunodominant VSG did not yield any universal protection. So far, attempts to immunize with trypanosome molecules (VSG, beta tubulin, etc.), such as conserved membrane proteins or receptors for uptake of nutrients, have resulted in limited protective effects because such molecules are either concealed beneath the surface coat or are expressed at a to low level to induce protective host immunity (140, 141). However, some protection against AT-associated pathological features (i.e., anemia, tissue injury) has been achieved upon vaccination with pathology-inducing factors such as the VSG-derived GPI moiety (*T. brucei, T. congolense*) or the cysteine proteinase congopain (*T. congolense*), yet the animals were never fully cured (142, 143). Moreover, as far as the GPI-based strategy in the murine model was concerned, there was no effect on parasitemia but rather the protective effect correlated with reduced pro-inflammatory immune responses and was independent of the Ab response. This is in line with the observation that pathogenicity did not correlate with Ab levels at least for the experimental murine *T. brucei* model (86). In contrast, for the congopain vaccination strategy in experimental bovine *T. congolense* models, there was a correlation between reduced pathogenicity and increased Ab titers (143, 144). This is in line with the observation that *T. congolense*-infected N’Dama cattle (a trypanotolerant breed showing natural resistance to trypanosomosis) exhibited higher antiparasite Ab titers than the susceptible Boran breeds (145, 146), suggesting that there are differences in the frequency of trypanosome-specific Ab-secreting cells in the spleen and in the activation state of B-cells in the blood between both cattle breeds during infection. Interestingly, the sera from *T. congolense*-infected N’Dama cattle specifically recognized dimer-associated epitopes on the congopain antigen (147).

The current research on vaccine development has switched toward identification of invariant surface glycoproteins or conserved *c*-terminal VSG epitopes/peptides that are predicted to contain several MHC II recognition sites (148–150). Whether these latter approaches will lead to the development of an effective protective and antipathology vaccine will be challenging and possibly not achievable given that African trypanosomes undermine B-cell memory responses. In addition, also the whole genome transcriptome analysis (i.e., SAGE technique) that enables to (i) explore the full transcriptome of trypanosusceptible and trypanotolerant cattle might lead to the identification of interesting gene variations linked to the trypanotolerance status of the animal (151–153) and (ii) understand the molecular aspects of the trypanosome dialog with its tsetse and mammalian hosts (i.e., interaction with the salivary glands and LS versus SS differentiation, respectively) might pave the way to develop novel diagnostic/therapeutic intervention strategies (154, 155). Furthermore, although the loss of B-cell responses/memory during AT might rely on either a parasite-induced or a host-induced effect or a combination of both, understanding the molecular mechanisms used by the trypanosomes to dampen B cell responses might lead to the development of new therapeutics not only for AT but also for other diseases such as autoimmune diseases (i.e., CIA) or malaria, where B-cell dysfunction is contributing to the disease outcome (131, 156–158). In this context, it was shown that the B-cell adaptor molecule Bam32 plays a pivotal role in optimal Ab responses and resistance during *T. congolense* infections in mice (159). Besides parasite-derived molecules, also host-derived molecule could be considered as a potential target for intervention strategies. In this context, MIF can be proposed as potential candidate given that it can play a role both in innate as adaptive immunity *via* interaction with its main receptor CD74 to regulate the host inflammatory response (160, 161). Indeed, MIF was shown to play a pivotal role in stimulating/inflammatory responses and regulating T- and B-cell recruitment as well as B-cell proliferation/survival and thereby contribute to pathology development (162–164). Hence, blocking MIF-signaling could reduce inflammatory responses, thereby alleviating suppression of B-cell lymphopoiesis, which in turn might favor vaccine efficacy.

In the future, most likely, a combination of (i) more sensitive/reliable diagnosis techniques needed for early-stage parasite detection and (ii) anti-parasite intervention strategies (trypanocidal/trypanotoxic drugs) and (iii) antidisease/pathology (anti-inflammatory) intervention strategies will be required to combat AT (see Figure 3).

## Author Contributions

All authors contributed to writing the manuscript. However, BS and MR are co-first and CT and SM share co-last authorship.

## Conflict of Interest Statement

The authors declare that the research was conducted in the absence of any commercial or financial relationships that could be construed as a potential conflict of interest. The reviewer, HG, and handling editor declared their shared affiliation, and the handling editor states that the process nevertheless met the standards of a fair and objective review.
